# Changes in medication safety indicators in England throughout the covid-19 pandemic using OpenSAFELY: population based, retrospective cohort study of 57 million patients using federated analytics

**DOI:** 10.1136/bmjmed-2022-000392

**Published:** 2023-05-11

**Authors:** Louis Fisher, Lisa EM Hopcroft, Sarah Rodgers, James Barrett, Kerry Oliver, Anthony J Avery, Dai Evans, Helen Curtis, Richard Croker, Orla Macdonald, Jessica Morley, Amir Mehrkar, Sebastian Bacon, Simon Davy, Iain Dillingham, David Evans, George Hickman, Peter Inglesby, Caroline E Morton, Becky Smith, Tom Ward, William Hulme, Amelia Green, Jon Massey, Alex J Walker, Christopher Bates, Jonathan Cockburn, John Parry, Frank Hester, Sam Harper, Shaun O’Hanlon, Alex Eavis, Richard Jarvis, Dima Avramov, Paul Griffiths, Aaron Fowles, Nasreen Parkes, Ben Goldacre, Brian MacKenna

**Affiliations:** 1 Bennett Institute for Applied Data Science, Nuffield Department of Primary Care Health Sciences, Oxford University, Oxford, UK; 2 PRIMIS, School of Medicine, Faculty of Medicine and Health Sciences, University of Nottingham, Nottingham, UK; 3 Centre for Academic Primary Care, School of Medicine, Faculty of Medicine and Health Sciences, University of Nottingham, Nottingham, UK; 4 TPP, Leeds, UK; 5 EMIS Health, Leeds, UK

**Keywords:** Primary health care, COVID-19, Medical informatics

## Abstract

**Objective:**

To implement complex, PINCER (pharmacist led information technology intervention) prescribing indicators, on a national scale with general practice data to describe the impact of the covid-19 pandemic on safe prescribing.

**Design:**

Population based, retrospective cohort study using federated analytics.

**Setting:**

Electronic general practice health record data from 56.8 million NHS patients by use of the OpenSAFELY platform, with the approval of the National Health Service (NHS) England.

**Participants:**

NHS patients (aged 18-120 years) who were alive and registered at a general practice that used TPP or EMIS computer systems and were recorded as at risk of at least one potentially hazardous PINCER indicator.

**Main outcome measure:**

Between 1 September 2019 and 1 September 2021, monthly trends and between practice variation for compliance with 13 PINCER indicators, as calculated on the first of every month, were reported. Prescriptions that do not adhere to these indicators are potentially hazardous and can cause gastrointestinal bleeds; are cautioned against in specific conditions (specifically heart failure, asthma, and chronic renal failure); or require blood test monitoring. The percentage for each indicator is formed of a numerator of patients deemed to be at risk of a potentially hazardous prescribing event and the denominator is of patients for which assessment of the indicator is clinically meaningful. Higher indicator percentages represent potentially poorer performance on medication safety.

**Results:**

The PINCER indicators were successfully implemented across general practice data for 56.8 million patient records from 6367 practices in OpenSAFELY. Hazardous prescribing remained largely unchanged during the covid-19 pandemic, with no evidence of increases in indicators of harm as captured by the PINCER indicators. The percentage of patients at risk of potentially hazardous prescribing, as defined by each PINCER indicator, at mean quarter 1 (Q1) 2020 (representing before the pandemic) ranged from 1.11% (age ≥65 years and non-steroidal anti-inflammatory drugs) to 36.20% (amiodarone and no thyroid function test), while Q1 2021 (representing after the pandemic) percentages ranged from 0.75% (age ≥65 years and non-steroidal anti-inflammatory drugs) to 39.23% (amiodarone and no thyroid function test). Transient delays occurred in blood test monitoring for some medications, particularly angiotensin-converting enzyme inhibitors (where blood monitoring worsened from a mean of 5.16% in Q1 2020 to 12.14% in Q1 2021, and began to recover in June 2021). All indicators substantially recovered by September 2021. We identified 1 813 058 patients (3.1%) at risk of at least one potentially hazardous prescribing event.

**Conclusion:**

NHS data from general practices can be analysed at national scale to generate insights into service delivery. Potentially hazardous prescribing was largely unaffected by the covid-19 pandemic in primary care health records in England.

**WHAT IS ALREADY KNOWN ON THIS TOPIC**

- Primary care services were substantially disrupted by the covid-19 pandemic
- Disruption to safe prescribing during the pandemic has not previously been evaluated
- PINCER is an evidence based, complex intervention to identify and correct hazardous prescribing in primary care; the intervention is pharmacist led and has been rolled out nationally to general practices in England

**WHAT THIS STUDY ADDS**

- This study is the most comprehensive assessment of medication safety during the covid-19 pandemic in England, covering 95% of the population using well validated indicators
- Good performance was maintained across many PINCER indicators throughout the pandemic
- Delays in delivering blood test monitoring for some medications were evident, although considerable recovery was made by the end of the study period

**HOW THIS STUDY MIGHT AFFECT RESEARCH, PRACTICE, OR POLICY**

- Collaborative working by openly sharing codelists and analytical code were beneficial
- Federated analytics has potential to provide near real time reporting on important public health issues at a national level

## Introduction

The World Health Organization (WHO) launched a patient safety challenge in 2017, Medication Without Harm,^1^ with an ambition to "reduce severe avoidable medication related harm globally by 50% in the next five years".^2^ The covid-19 pandemic disrupted the delivery of primary care services within the National Health Service (NHS) in the UK from mid-March 2020, with a reduction of 30% in general practitioner (GP) consultations, 74% in routine referrals, and 43% in urgent cancer referrals, compared with precovid baselines.^3 4^ The extent of disruption varied by clinical context,^5–7^ although most primary care services were restored by September 2020.^8 9^ The disruption during this time might have contributed towards increased rates of harm related to medication, with 34% of an estimated 66 million potentially clinically significant errors occurring in primary care prescribing in England annually, as estimated by NHS dispensing statistics in 2015-16.^10^

As part of its response to WHO's challenge, PRIMIS at the University of Nottingham led on the national roll-out of PINCER (pharmacist-led information technology intervention for medication errors) in collaboration with the Academic Health Science Networks.^11^ The PINCER intervention is a proven programme of activities for reducing hazardous prescribing in general practices (more information provided in online supplemental text).^12^ Briefly, the intervention involves training pharmacists working in general practice to provide feedback, educational outreach, and dedicated support, systematically focusing on patients who are identified to be at risk of harm from medications. These patients are identified using prespecified and quality assured analytical indicators in the Systematized Nomenclature of Medicine Clinical Terms SNOMED-CT code classification system used by general practice systems in England. PINCER includes 13 indicators of hazardous prescribing of high risk medications prescribed in primary care that: (1) can cause gastrointestinal bleeds; (2) are cautioned against in specific conditions (heart failure, asthma, and chronic renal failure); or (3) require blood test monitoring (box 1). These indicators have been developed from collaboration between academics from the University of Nottingham and made available to pharmacists in practices participating in the PINCER programme (online supplemental text).

10.1136/bmjmed-2022-000392.supp1 Supplementary data

Box 1 **The 13 PINCER indicators (shortened terms used hereafter)**

**Prescribing indicators associated with gastrointestinal bleeding**

- Oral non-steroidal anti-inflammatory drug (NSAID), without co-prescription of an ulcer healing drug, to a patient of ≥65 years (age ≥65 years and NSAID)
- Oral NSAID, without co-prescription of an ulcer healing drug, to a patient with a history of peptic ulceration (peptic ulceration and NSAID)
- Antiplatelet drug, without co-prescription of an ulcer healing drug, to a patient with a history of peptic ulceration (peptic ulceration and antiplatelet)
- Warfarin or direct oral anticoagulants (DOAC) in combination with an oral NSAID (warfarin or DOAC and NSAID)
- Warfarin or DOAC and an antiplatelet drug, without co-prescription of an ulcer healing drug (warfarin or DOAC)
- Aspirin in combination with another antiplatelet drug, without co-prescription of an ulcer healing drug (aspirin and other antiplatelet)

**Prescribing indicators associated with cautioned medication in other conditions**

- Oral NSAID to a patient with heart failure (heart failure and NSAID)
- Non-selective beta blocker to a patient with asthma (asthma and beta blocker)
- Oral NSAID to a patient with estimated glomerular filtration rate of <45 (chronic renal failure and NSAID)

**Prescribing indicators associated with blood test monitoring**

- Long term prescription of angiotensin-converting enzyme inhibitor or a loop diuretic to patients aged ≥75 years who have not had a computer recorded check of their renal function and electrolytes in the previous 15 months (angiotensin-converting enzyme inhibitor or a loop diuretic, no blood tests)
- Methotrexate treatment for at least three months in people who have not had a recorded:
  - Full blood count within the previous three months (methotrexate and no full blood count); or
  - Liver function test within the previous three months (methotrexate and no liver function test)
- Lithium treatment for at least three months in people who have not had a recorded check of their lithium concentrations in the previous three months (lithium and no level recording)
- Amiodarone treatment for at least six months who have not had a thyroid function test within the previous six months (amiodarone and no thyroid function test)

OpenSAFELY is a secure analytics platform for electronic patient records built by our group with the approval of NHS England to deliver urgent academic^13^ and operational NHS service research^14 15^ on the direct and indirect effects of the pandemic. Analyses can use patients’ full, raw, pseudonymised primary care records at 95% of English general practices (55% use EMIS software, and 40% use TPP software) with patient level linkage to various sources of secondary care data. All code and analysis is shared openly for inspection and re-use.

The PINCER indicators created by PRIMIS are typically implemented for single practices, or groups of practices, through various technical methods (online supplemental materials) in different settings to monitor compliance for practices that are participating in the PINCER programme. We aimed to implement the full suite of PINCER codelists, methods, and indicators in OpenSAFELY to allow monitoring of compliance on all prescribing safety indicators at a population level. Additionally, we aimed to describe changes in compliance after the disruption induced by covid-19 to primary care services in England.

## Methods

### Study design

We conducted a retrospective cohort study using general practice primary care electronic health record data from all GP practices in England, supplied by the electronic health record vendors TPP and EMIS.

### Data source

Primary care records managed by the GP software providers TPP and EMIS are available in OpenSAFELY, a data analytics platform created by our team with the approval of NHS England to address urgent covid-19 research questions (https://opensafely.org). OpenSAFELY provides a secure software interface allowing the analysis of pseudonymied primary care patient records from England in near real-time within the electronic health record vendor’s highly secure data centre. This interface avoids the need for large volumes of potentially disclosive, pseudonymised patient data to be transferred off-site. Therefore, in addition to other technical and organisational controls, any risk of re-identification is minimised. Similarly pseudonymised datasets from other data providers are securely provided to the electronic health record vendor and linked to the primary care data. The TPP dataset analysed within OpenSAFELY (hereafter OpenSAFELY-TPP) is based on 24.2 million people registered with 2546 GP surgeries using the TPP SystmOne software. The EMIS dataset analysed within OpenSAFELY (hereafter OpenSAFELY-EMIS) is based on 32.6 million people registered with 3821 GP surgeries using EMIS. These datasets contain pseudonymised data such as coded diagnoses and physiological parameters, including blood test results requested by the practice. OpenSAFELY makes extensive use of electronic health record data regarding primary care prescriptions in the NHS in England. Briefly, health professionals who are able to write prescriptions in primary care in the NHS in England include GPs, suitably qualified nurses, pharmacists, and physiotherapists. With very few exceptions, every prescription written by these prescribers is recorded within the patient’s record in general practice clinical systems. These prescriptions are then dispensed by a dispensing service commissioned by the NHS, usually a community pharmacy or dispensing doctor in rural locations. No free text data are included. Further details can be found later in the information governance and ethical approval sections. More information about the platform is available on OpenSAFELY's website; in our review for the UK Government's Department of Health and Social Care^16^; and in our previous publications.^13^

### Study population

We included patients who were: alive, aged 18-120 years, registered with an OpenSAFELY-TPP or OpenSAFELY-EMIS practice, and recorded as at risk of at least one potentially hazardous prescribing indicator. A patient was considered as being at risk if they were categorised into at least one of the PINCER indicator denominators as defined by the SNOMED-CT)[Bibr R17],^17^ [Bibr R17]NHS dictionary of medicines and devices (dm+d) codes,^17^ and associated logic developed by PRIMIS for the PINCER programme, as assessed on the first of each month between 1 September 2019 and 1 September 2021, inclusive. This time period was chosen to adequately cover the period of service disruption onset and subsequent service recovery due to the covid-19 pandemic in the UK.

### Study measures

Definitions of the hazardous prescribing indicators are described in box 1. The percentage for each indicator is formed of a numerator, which captures patients deemed by the indicator to be at risk of a potentially hazardous prescribing event and a denominator, which captures all patients for which assessment of the indicator is clinically meaningful. Higher indicator percentages represent potentially poorer performance on medication safety (online supplemental file 1). Indicators belong to one of three groups: those associated with gastrointestinal bleeds; those associated with cautioned medications; and those associated with blood test monitoring.

We specified each indicator in analytical code using PRIMIS SNOMED-CT codelists^17^ and the OpenSAFELY framework. We generated the numerator and denominator for each indicator every month between 1 September 2019 and 1 September 2021, and then calculated monthly percentages for each practice. For indicators assessing numerical values, only unambiguous results were used in the calculation of indicator percentages (eg, an estimated glomerular filtration rate value of >30 was considered ambiguous for an indicator requiring the identification of patients with a rate of <45). Note that this functionality was not available in OpenSAFELY-EMIS at the time of the study, therefore, results for the chronic renal failure and NSAID indicator are reported for OpenSAFELY-TPP practices only.

The monthly indicator percentages were summarised as deciles and presented as decile charts across all practices each month. We also calculated the mean rate across practices in quarter 1 (Q1) 2020 and 2021 for each indicator as well as total counts of the numerator and denominator for each indicator across the two years. Note that in these cumulative data, repeated events will be counted for each month the event occurs (eg, if a patient with heart failure is prescribed an oral NSAID in two separate months, this action is represented as two separate events). Across this period, we also calculated the ratio of hazardous prescribing events to unique patients who had those events (to give an indication of the extent of repeated hazardous prescribing) and the number and percentage of practices with at least one instance of potentially hazardous prescribing at any point across the period.

Each blood test monitoring indicator has an associated monitoring window (eg, lithium concentrations are required to be checked within three months). Should no action have been taken to rectify covid-19 related delays, then 100% of patients will have had delayed blood test monitoring by the end of the relevant monitoring window. For each blood test monitoring indicator, we have calculated the projected month of maximum impact from the onset of covid-19 related disruption in March 2020 (ie, June 2020 for the methotrexate and lithium monitoring indicators, September 2020 for the amiodarone monitoring indicator, and May 2021 for the ACE inhibitor monitoring indicator). Each decile plot has been annotated to indicate when this projected date of maximum impact would have occurred.

### Software and reproducibility

For data management and analysis, we used the OpenSAFELY software libraries and Python (Python 3.8). A federated analysis involves carrying out patient level analysis in multiple secure datasets, then later combining them. Codelists and code for data management and data analysis were specified once using the OpenSAFELY tools; then transmitted securely to the OpenSAFELY-TPP platform within TPP’s secure environment, and separately to the OpenSAFELY-EMIS platform within EMIS’s secure environment, where they were each executed separately against local patient data. Summary results were then reviewed to assess the potential for patient or practice re-identification before being released and combined for the final outputs. All code for the OpenSAFELY platform for data management, analysis, and secure code execution is shared for review and re-use under open licences at github.com/OpenSAFELY. Decile charts were drawn using Seaborn and matplotlib.

### Information governance

NHS England is the data controller for OpenSAFELY-EMIS and OpenSAFELY-TPP; EMIS and TPP are the data processors; all study authors using OpenSAFELY have the approval of NHS England. This implementation of OpenSAFELY is hosted within the EMIS and TPP environments, which are accredited to the ISO 27001 information security standard and are are Data Security and Protection Toolkit compliant.^18^

Patient data have been pseudonymised for analysis and linkage using industry standard cryptographic hashing techniques and all pseudonymised datasets transmitted for linkage onto OpenSAFELY are encrypted. Access to the platform is via a virtual private network connection, restricted to a small group of researchers; the researchers hold contracts with NHS England and only access the platform to initiate database queries and statistical models. All database activity is logged and only aggregate statistical outputs leave the platform environment following best practice for anonymisation of results, such as statistical disclosure control for low cell counts.^19^

The OpenSAFELY research platform adheres to the obligations of the UK General Data Protection Regulation (known as GDPR) and the Data Protection Act 2018. In March 2020, the Secretary of State for Health and Social Care used powers under the UK Health Service (Control of Patient Information) Regulations 2002 (known as COPI) to require organisations to process confidential patientinformation for the purposes of protecting public health, providing healthcare services to the public, and monitoring and managing the covid-19 outbreak and incidents of exposure; thereby removings the requirement for patient consent.^20^ This regulation was extended in November 2022 for the NHS England OpenSAFELY covid-19 research platform.^21^ In some cases of data sharing, the common law duty of confidence is met using, for example, patient consent or support from the Health Research Authority Confidentiality Advisory Group.^22^

Taken together, these provide the legal bases to link patient datasets on the OpenSAFELY platform. GP practices, from which the primary care data are obtained, are required to share relevant health information to support the public health response to the pandemic, and have been informed of the OpenSAFELY analytics platform.

### Patient and public involvement

We have involved patients and the public in various ways: we developed a public website that provides a detailed description of the platform in language suitable for a lay audience; we have participated in two citizen juries exploring public trust in OpenSAFELY^23^; we are co-developing an explainer video; we have patient representation who are experts by experience on our OpenSAFELY Oversight Board; we have partnered with Understanding Patient Data to produce lay explainers on the importance of large datasets for research; we have presented at various online public engagement events to key communities (eg, Healthcare Excellence Through Technology; Faculty of Clinical Informatics annual conference; NHS Assembly; HDRUK symposium); and more. To ensure the patient voice is represented, we are working closely to decide on language choices with appropriate medical research charities (eg, Association of Medical Research Charities). We will share information and interpretation of our findings through press releases, social media channels, and plain language summaries.

## Results

We identified 1 813 058 (3.1% of 56.8 million) patients registered across 6367 practices who were at risk of potentially hazardous prescribing, as indicated by the PINCER indicators, at any point between 1 September 2019 and 1 September 2021. Demographic characteristics for the 14 284 444 (25.1%) patients who were identified in at least one indicator denominator in the last month of the study period (September 2021) are provided in table 1.

**Table 1:** Cohort description for any patients included in the denominator of at least one of the PINCER indicators at the end of the study period (September 2021), in OpenSAFELY-TPP and OpenSAFELY-EMIS

Category	OpenSAFELY-TPP	OpenSAFELY-EMIS	Total
**Overall**			
Total	5 998 805 (100.00)	8 285 639 (100.00)	14 284 444 (100.00)
**Age**			
18-19	69 947 (1.17)	93 636 (1.13)	163 583 (1.15)
20-29	473 181 (7.89)	679 780 (8.20)	1 152 961 (8.07)
30-39	507 839 (8.47)	788 895 (9.52)	1 296 734 (9.08)
40-49	468 532 (7.81)	689 394 (8.32)	1 157 926 (8.11)
50-59	527 714 (8.80)	766 127 (9.25)	1 293 841 (9.06)
60-69	1 217 103 (20.29)	1 647 347 (19.88)	2 864 450 (20.05)
70-79	1 672 710 (27.88)	2 235 918 (26.99)	3 908 628 (27.36)
≥80	1 061 779 (17.70)	1 384 542 (16.71)	2 446 321 (17.13)
**Sex**			
Female	3 122 219 (52.05)	4 291 110 (51.79)	7 413 329 (51.90)
Male	2 876 586 (47.95)	3 994 529 (48.21)	6 871 115 (48.10)
**Ethnicity**			
White	4 285 420 (71.44)	5 480 579 (66.15)	9 765 999 (68.37)
South Asian	209 386 (3.49)	405 580 (4.89)	614 966 (4.31)
Black	69 225 (1.15)	199 570 (2.41)	268 795 (1.88)
Other	48 206 (0.80)	77 498 (0.94)	125 704 (0.88)
Mixed	38 392 (0.64)	84 486 (1.02)	122 878 (0.86)
Missing	1 348 176 (22.47)	2 037 926 (24.60)	3 386 102 (23.70)
**Index of Multiple Deprivation**			
1 (most deprived)	984 981 (16.42)	1 425 367 (17.20)	2 410 348 (16.87)
2	1 094 257 (18.24)	1 569 287 (18.94)	2 663 544 (18.65)
3	1 306 389 (21.78)	1 649 173 (19.90)	2 955 562 (20.69)
4	1 283 629 (21.40)	1 741 211 (21.01)	3 024 840 (21.18)
5 (least deprived)	1 227 902 (20.47)	1 875 295 (22.63)	3 103 197 (21.72)
Missing	101 647 (1.69)	25 306 (0.31)	126 953 (0.89)
**Region**			
East	1 337 146 (22.29)	343 151 (4.14)	1 680 297 (11.76)
London	276 220 (4.60)	1 431 617 (17.28)	1 707 837 (11.96)
Midlands*	1 282 238 (21.37)	1 494 269 (18.03)	2 776 507 (19.44)
North East and Yorkshire†	1 548 534 (25.81)	702 833 (8.48)	2 251 367 (15.76)
North West	94 912 (1.58)	1 693 016 (20.43)	1 787 928 (12.52)
South East	502 990 (8.38)	1 905 796 (23.00)	2 408 786 (16.86)
South West	956 765 (15.95)	714 957 (8.63)	1 671 722 (11.70)

Data are number (percentage).

*Comprised of East Midlands and West Midlands in OpenSAFELY-TPP.

†Comprised of Yorkshire and the Humber and North East in OpenSAFELY-TPP.

For each PINCER indicator, we show Q1 mean percentages for 2020 (ie, a precovid-19 onset period) and 2021 (ie, a postcovid-19 onset period) to enable comparison of service disruption related to before and after covid-19 (table 2). Mean Q1 2020 percentages ranged from 1.11% (age ≥65 years and NSAID) to 36.20% (amiodarone and no thyroid function test), while Q1 2021 percentages ranged from 0.75% (age ≥65 years and NSAID) to 39.23% (amiodarone and no thyroid function test). The difference of before and after covid-19 onset ranged from a reduction of 0.59% (warfarin/DOAC and antiplatelet) to an increase of 6.98% (angiotensin-converting enzyme inhibitors or loop diuretic and no blood tests).

**Table 2:** Indicator rates for PINCER hazardous prescribing indicators: Q1 2020-21 percentages and cumulative results between 1 September 2019 and 1 September 2021

Indicator	Q1 2020 mean percentage	Q1 2021 mean percentage	Cumulative counts		
			Numerator/denominator (%)	Ratio of hazardous prescribing events to unique patients having an event	Practices with ≥1 hazardous prescribing events (% of total practices)
Gastrointestinal bleeding	—	—	551 844/10 881 675 (5.07)	5.53	6335 (99.50)
Age ≥65 years and NSAID	1.11	0.75	334 487/9 207 007 (3.63)	4.54	6304 (99.01)
PU and NSAID	1.32	1.07	32 089/678 218 (4.73)	3.83	5801 (91.11)
PU and antiplatelet	4.24	3.85	41 414/678 218 (6.11)	10.90	5943 (93.34)
Warfarin/DOAC and NSAID	1.39	1.18	84 101/1 915 117 (4.39)	4.57	6193 (97.27)
Warfarin/DOAC and antiplatelet	2.26	1.67	52 575/1 249 865 (4.21)	5.86	6068 (95.30)
Aspirin and other antiplatelet	1.67	1.20	36 927/1 470 315 (2.51)	7.15	5777 (90.73)
Cautioned medications	—	—	228 587/7 594 687 (3.01)	9.43	6321 (99.28)
HF and NSAID	1.71	1.43	31 034/735 781 (4.22)	6.09	5622 (88.30)
Asthma and beta blocker	1.27	1.29	185 289/6 646 586 (2.79)	10.24	6307 (99.06)
CRF and NSAID	1.27	1.12	14 558/476 925 (3.05)	4.70	2217 (87.08)
Blood test monitoring	—	—	1 102 209/3 358 954 (32.81)	6.59	6329 (99.40)
ACEI or loop diuretic, no blood tests	5.16	12.14	850 587/3 095 595 (27.48)	5.89	6303 (98.99)
Methotrexate and no FBC	18.64	22.73	164 502/238 042 (69.11)	5.05	6278 (98.60)
Methotrexate and no LFT	19.62	23.27	166 484/238 042 (69.94)	5.14	6278 (98.60)
Lithium and no level recording	31.47	38.44	40 664/45 456 (89.46)	6.93	5912 (92.85)
Amiodarone and no TFT	36.20	39.23	46 268/59 925 (77.21)	6.22	5993 (94.13)

ACEI=angiotensin-converting enzyme inhibitors; CRF=chronic renal failure; DOAC=direct oral anticoagulants; FBC=full blood count; HF=heart failure; LFT=liver function test; NSAID=non-steroidal anti-inflammatory drugs; PU=peptic ulceration; TFT=thyroid function test. Mean values are calculated at the practice level; higher mean values indicate increased rates of potentially hazardous prescribing across practices in the period. Rates for CRF and NSAID are calculated across 2546 OpenSAFELY-TPP practices; all other indicator rates are calculated across all 6367 practices (2546 OpenSAFELY-TPP plus 3821 OpenSAFELY-EMIS practices). Tables for OpenSAFELY-TPP and OpenSAFELY-EMIS separately are available in online supplemental tables 1 and 2.

Cumulative counts for each indicator are provided in table 2. The percentage of patients identified as at risk of a potentially hazardous prescribing event in the study period ranged from 2.51% (36 927 of 1 470 315 patients for aspirin and other antiplatelet) to 89.46% (40 664 of 45 456 patients for lithium and no level recording). The ratio of hazardous events to patients ranged from 3.83 (peptic ulceration and NSAID) to 10.90 (peptic ulceration and antiplatelet). The percentage of practices with an event for each indicator ranged from 87.02% (chronic renal failure and NSAID) to 99.06% (asthma and beta blocker).

### Indicators associated with gastrointestinal bleeding

**Figure 1:**
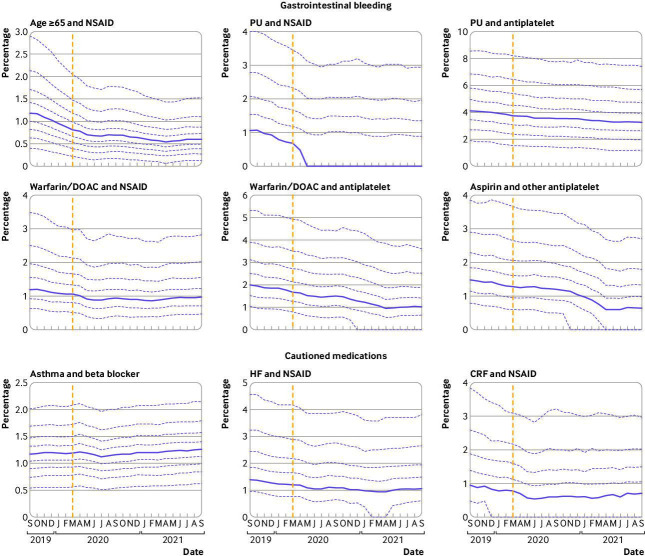
Practice level decile plots for PINCER prescribing indicators, specifically in relation to (top two rows) gastrointestinal bleeding and (bottom row) cautioned medications. The percentage of patients identified as at risk of potentially hazardous prescribing as measured by each indicator is reported by month. Months are displayed on on the x-axis, starting from September 2019 to September 2021. The median percentage is displayed as a thick purple line and deciles are indicated by dashed purple lines. The month of national lockdown in England as a response to the onset of covid-19 (March 2020) is highlighted with a yellow dashed vertical line. Deciles for CRF and NSAID are calculated across 2546 OpenSAFELY-TPP practices; all other deciles are calculated across 6367 practices (2546 OpenSAFELY-TPP practices plus 3821 OpenSAFELY-EMIS practices). Decile plots for these same indicators, in OpenSAFELY-TPP and OpenSAFELY-EMIS separately, are available in online supplemental figures 1 and 2, respectively. CRF=chronic renal failure; DOAC=direct oral anticoagulants; HF=heart failure; NSAID=non-steroidal anti-inflammatory drugs; PU=peptic ulceration

The six indicators of potentially hazardous prescribing in relation to gastrointestinal bleeds decreased across the study period with no evidence of an increase in hazardous prescribing as a result of covid-19 induced service disruption (figure 1 (top two rows), OpenSAFELY-TPP only and OpenSAFELY-EMIS only decile charts are provided in online supplemental figures 1A and 2A, respectively). The mean percentage of warfarin/DOAC and NSAID was 1.39% in Q1 2020; the equivalent rate in 2021 was 1.18%. Similarly, the percentage of peptic ulceration and antiplatelet reduced from 4.24% in Q1 2020 to 3.85% in Q1 2021 (table 2). This improving trend was only marginally impacted during the months of the greatest service disruption due to the covid-19 pandemic. Gastrointestinal bleed indicators had lower incidence among some practices than others. For example, the percentage of practices that had at least one hazardous prescribing event for the aspirin and other antiplatelet indicator was 90.73% and for age ≥65 years and NSAID indicator was 99.01%.

### Indicators associated with cautioned medications

Time trends and variation for all three cautioned medication indicators are presented in figure 1 (bottom row) (OpenSAFELY-TPP only and OpenSAFELY-EMIS only decile charts are provided in online supplemental figures 1b and 2b, respectively). No evidence suggests that the covid-19 related disruption to service delivery had any substantial effect on compliance for all indicators associated with cautioned medications. Notably lower incidence of practices that had at least one hazardous prescribing event were reported for the chronic renal failure and NSAID indicator and for the heart failure and NSAID indicator with 87.08% and 88.30% of practices, respectively, reporting at least one hazardous prescribing event; in comparison, the asthma and beta blocker indicator had 99.06% of practices that had at least one hazardous prescribing event.

### Indicators associated with blood test monitoring

**Figure 2:**
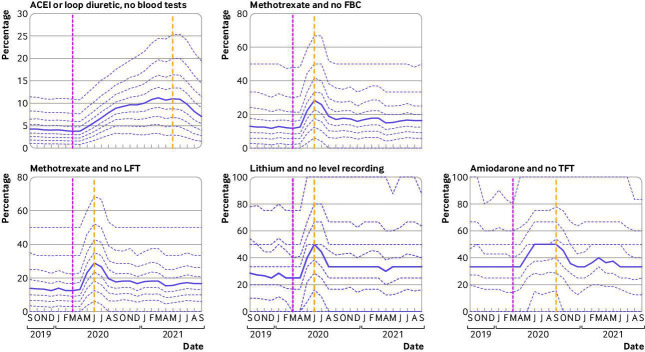
Practice level decile plots for PINCER blood test monitoring indicators. The percentage of patients identified as at risk of potentially hazardous prescribing as measured by each indicator is reported for the period September 2019 to September 2021 (inclusive). The median percentage is displayed as a thick purple line and deciles are indicated by dashed purple lines. The month of national lockdown in England as a response to the onset of covid-19 (March 2020) is highlighted with a pink dashed vertical line. The project date of maximum impact, as measured from the onset of covid-19, for each indicator is shown by a yellow dashed vertical line. All deciles are calculated across 6367 practices (2546 OpenSAFELY-TPP plus 3821 OpenSAFELY-EMIS practices). Decile plots for these same indicators, in OpenSAFELY-TPP and OpenSAFELY-EMIS separately, are available in online supplemental figures 3 and 4 respectively. ACEI=angiotensin-converting enzyme inhibitors; FBC=full blood count; LFT=liver function test; TFT=thyroid function test

All blood test monitoring indicators exhibited an increase in delayed monitoring immediately after the onset of covid-19 (May-July 2020; figure 2). These increased rates showed considerable recovery by August-September 2020, in the case of lithium and no level recording (31.47% Q1 2020 *v* 38.44% Q1 2021), methotrexate and no full blood count (18.64% *v* 22.73%), and methotrexate and no liver function test (19.62% *v* 23.27%). The amiodarone and no thyroid function test indicator had similar results (36.20% *v* 39.23%), although the initial recovery period after covid-19 extended into October-November 2020 (table 2). As with the other groups of indicators, the incidence among practices is high, although this value varies by indicator: incidence varies from 92.85% (lithium and no level recording) to 98.99% (angiotensin-converting enzyme inhibitors or loop diuretic, no blood tests).

The indicator of angiotensin-converting enzyme inhibitors or loop diuretic and no blood tests had a noticeably different covid-19 response pattern than the other blood test monitoring indicators. Here,the monitoring worsened steadily over a longer period of time, increasing from a mean of 5.16% to 12.14% between Q1 2020 and Q1 2021, and began to recover in June 2021. The assessment window for this indicator is substantially wider than the windows for the other blood test monitoring indicators: within 15 months of prescription compared with three months for lithium and methotrexate or six months for amiodarone.

## Discussion

### Summary

Despite substantial barriers to the delivery of primary care during the covid-19 pandemic, good performance was maintained across a diverse range of widely evaluated and nationally adopted indicators of safe prescribing. Delays were evident in delivering some medication related blood test monitoring within the time window specified in the safety measure; especially for those blood tests where the time window for compliance is already very long, and tests infrequent (specifically the angiotensin-converting enzyme inhibitor or loop diuretic indicator). However, all indicators exhibited considerable recovery by the end of the study period.

### Strengths and limitations

This study has a range of strengths. Compared with other routes of access to primary care data, OpenSAFELY offers a more complete coverage of more patients with greater controls on security and complete transparency in terms of the method and reproducibility. Previous audits for compliance with PINCER or similar measures and indicators in primary care rely on manual audit within a practice, or analyses of data downloaded from a group of practices. By contrast, OpenSAFELY executes analyses in a secure environment inside the electronic health record provider data centre, across the full set of all structured data in the GP record including all tests, prescriptions, diagnostic codes, and referrals. Additionally, although the underlying GP data are stored in two very different settings (TPP and EMIS), PINCER indicators were described for almost all GP practices (about 99%) in England for the first time by use of a single analysis in OpenSAFELY. The necessary variables and analyses were defined once, then executed in each setting identically, with the outputs aggregated afterwards, in a process known as federated analytics. Overall, this national platform is uniquely able to capture the patient journey for 57 million people in England while prioritising patient privacy.

Another strength is the transparency and reproducibility of the analysis. OpenSAFELY openly shares on GitHub all code for the platform, data curation, and analysis, from raw data to completed output. These data are in standard formats for scientific review and efficient re-use under open licences by all.

Additionally, the indicators for each safety behaviour are robust. All eligible patients and targeted clinical safety behaviours were developed for the national PINCER medication safety programme. This programme has been extensively peer reviewed and evaluated throughout the NHS over many years, with strong support from clinicians and commissioners.

We also note some limitations. Our results are only descriptive in nature: we have not attempted to statistically assess the extent to which indicator rates changed during the period of service disruption after the onset of covid-19, or the extent to which prepandemic rates were recovered. Furthermore, our study period does not allow for consideration of a time before the pandemic in which variation in indicator rates over time could inform such statistical hypothesis testing. Finally, we acknowledge that our data will only include prescriptions and test results carried out in primary care, or those in secondary care that are returned to GPs as structured data. As such, these data might not include test results communicated by letter or phone (eg, tests requested while a person is in hospital or psychiatric outpatients). However, this method is aligns with others already used in the national PINCER programme to evaluate compliance with the targeted safety behaviours using primary care data alone.

### Comparison of existing literature

A systematic review of healthcare usage during the pandemic, encompassing 81 studies across 20 countries, found that healthcare use (eg, visits, admissions, diagnostics, and therapeutics) reduced by 37% during the pandemic, highlighting a substantial reduction in April-May 2020.^24^ WHO also identified substantial disruption to countries' healthcare capacity for non-communicable diseases in a rapid assessment in May 2020.^25^ A population based cohort study conducted using the OpenSAFELY platform reported that clinical activity in relation to blood tests declined in the months after covid-19 onset, but also reported recovery of these same tests by September 2020.^8^ These findings concord with our observations of the blood test monitoring indicators, where delays were substantial in the same period of time. Interrupted service delivery leading to reduced NSAID prescriptions after acute presentations might also explain the temporary reduction of the first gastrointestinal bleed indicator (prescription of an oral NSAID, without co-prescription of an ulcer healing drug, to a patient of ≥65 years) in April-July 2020. This finding is supported by data for this period in OpenPrescribing^26^ and lower than predicted rates of prescribing for naproxen and ibuprofen in this period.^27^ Elsewhere, we have found evidence of prioritisation of anticoagulant services, with blood tests to manage high risk anticoagulants being prioritised during the initial stages of the covid-19 pandemic.^8^ Data from our study also suggest that prescribing in relation to anticoagulants is a priority, with all gastrointestinal bleed indicators being unaffected, and continuing to decline, after covid-19 onset.

In the early stages of the pandemic, in recognition of the increased risk of medication related harm during the covid-19 pandemic, NHS England and local Clinical Commissioning Groups (known as CCGs) revised guidance regarding blood test monitoring. They extended the recommended monitoring window for some patient populations (eg, in relation to lithium^28^ and methotrexate^29^) or advised to monitor blood for lower risk medications if possible (eg, ACE inhibitors^30^), if clinically safe to do so. Some evidence in our data suggests that practices did adopt this revised guidance, with postrecovery blood test monitoring often less than prepandemic levels (particularly in the case of methotrexate and lithium).

### Implications for policy and research

The variation in service recovery observed in the blood test monitoring indicators might partly be due to the assessment window for each indicator, and clinicians prioritising urgent work during the pandemic. For example, the protracted recovery of the ACE inhibitor monitoring was possibly due to primary care services proactively prioritising monitoring of higher risk prescriptions, such as methotrexate, so as to minimise the impact of service disruption on patient care. The systems around the monitoring of high risk drugs (eg, clinical system alerts) also probably contributed towards expedited recovery of the other blood test monitoring indicators, particularly in the case of lithium and methotrexate. The decile chart for this indicator starts to plateau well before the ‘worst case scenario’ time point, suggesting that most primary care providers successfully implemented recovery programmes in this clinical domain. Further areas for research include using innovative change detection methods^31^ to ascertain practice level features that affect recovery and resilience in the context of service disruption to inform WHO and NHS England’s recommendations to build back better.

The potential impact of this analysis for data usage in the NHS is considerable. Historically, as a result of practical and privacy challenges around accessing GP data at scale. Each practice participating in the PINCER programme has been required to manually execute the necessary computerised searches before individually uploading their results for central oversight; in some centres, data for a group of practices and patients can be downloaded and analysed in larger volumes. This manual approach introduces delays and increases the resource cost of monitoring safety. Using the OpenSAFELY framework, we were able to execute a single analysis for almost the entire population of England in near real time, while leaving data in situ. This approach is efficient: analyses can be easily updated, and expanded, because they are executed in a single framework from re-executable code. Patient trust is also preserved: OpenSAFELY was the single most highly trusted covid-19 data project in a rigorous Citizens Jury sponsored by the NHS and the National Data Guardian.^23^ Furthermore, the additional data that are also securely accessible through the OpenSAFELY tools can be used to describe PINCER indicators in fine grained demographic or clinical subpopulations. These tools can facilitate near real time audit and feedback in the context of rapidly evolving pressures on the health service and are readily extendable to other clinical and challenges.

More broadly, this analysis shows the benefits of collaborative working with shared open source code in the NHS: it built on the work of PRIMIS in establishing and then publicly releasing the full code for a set of rigorously tested medication safety indicators; and then implemented the open code from PINCER in the open source framework of OpenSAFELY, to assess a critical public health question on a national scale. Open working. as shown here. is strongly supported by senior stakeholders in multiple sectors^16 32 33^ and can bring substantial benefits. Open working facilitates efficient re-use of previous technical work; ensures fidelity through the consistent implementation of data curation and analysis across all organisations; supports complete reproducibility; enables error checking by all interested parties; and facilitates public and professional trust.

### Conclusion

NHS GP data can be analysed at national scale to generate insights on service delivery. Potentially hazardous prescribing was largely unaffected by covid-19 in a dataset of 57 million patients’ full primary care health records in England.

## Data Availability

Data may be obtained from a third party and are not publicly available. We rapidly delivered the OpenSAFELY data analysis platform without prior funding to deliver timely analyses on urgent research questions in the context of the global Covid-19 health emergency: now that the platform is established we are developing a formal process for external users to request access in collaboration with NHS England; details of this process are available at OpenSAFELY.org/onboarding-new-users.
