# Supplementary material for: Changes in medication safety indicators in England throughout the covid-19 pandemic using OpenSAFELY: population based, retrospective cohort study of 57 million patients using federated analytics
Source: BMJ Med. 2023 May 11;2(1):e000392. doi: 10.1136/bmjmed-2022-000392 (PMC10254692; doi:10.1136/bmjmed-2022-000392)
Supplement: Supplementary data [file bmjmed-2022-000392supp001.pdf]

PINCER Intervention

The PINCER intervention was designed to (1) identify and (2) reduce rates of potentially hazardous prescribing[1,2][1] Over the last three years, PRIMIS—a unit of the University of Nottingham providing expert advice on the intelligent use of primary care data to the NHS, academics, and industry partners—has led on the national rollout of PINCER in collaboration with the Academic Health Science Network [3].

The PINCER intervention is comprised of three components. The first component of the intervention is a set of **prescribing safety indicators** [3], which are used to search GP computer systems to identify patients at risk of potentially hazardous prescribing. These searches can be deployed in a number of different ways. For example they can be deployed at an individual practice level or for a group of practices at the same time (e.g. using EMIS Enterprise solution); they can be deployed by members of the practice team or (with the correct permissions in place) be deployed remotely by PRIMIS on behalf of the practice. They can also be deployed using a third party solution. Patients lists are then generated where their prescriptions potentially do not adhere to the prescribing safety guidance; aggregating these scores allows calculation of compliance against an indicator of potentially unsafe prescribing for the whole practice. Indicators belong to three groups: (1) those associated with gastrointestinal bleeds; (2) those associated with cautioned medications; and (3) those associated with blood test monitoring. It is this component of the PINCER intervention that is immediately relevant to the current study.

The remaining two components of the PINCER intervention, which ensure that improved outcomes are achieved for patients, are:

1. Pharmacists, specifically trained to deliver the intervention, providing an educational outreach intervention where they meet with GPs and other practice staff to:
    a. Discuss the search results and highlight the importance of the hazardous prescribing identified using brief educational materials
    b. Agree an action plan for reviewing patients identified as high risk and improving prescribing and medication monitoring systems using root cause analysis (RCA) to minimise future risk
2. Pharmacists (and pharmacy technicians) working with, and supporting, general practice staff to implement the agreed action plan.

Findings from the PINCER trial, published in the Lancet [2], demonstrated that PINCER is an effective and cost-effective method for reducing a range of clinically important and commonly made medication errors in primary care. For example, at 6 months' follow-up, patients in the PINCER group were significantly less likely to have been prescribed an oral non-steroidal anti-inflammatory drug (NSAID) if they had a history of peptic ulcer without gastroprotection (Odds ratio (OR): 0·58; 95% Confidence Interval (CI): 0·38–0·89), thereby reducing their risk of hospital admission with gastrointestinal (GI) bleeding.

It is important to appreciate that the PINCER indicators capture rates of *potentially* hazardous prescribing: in some scenarios there may be a legitimate reason for a GP not to comply with

the prescribing behaviour suggested by the indicator (e.g., in the case of the *Asthma & beta-blocker* indicator, it may be necessary to prescribe a beta-blocker to a patient with substantial cardiovascular disease and/or allergies to other cardiovascular disease medication despite having a recorded diagnosis of asthma). In this context, full compliance is not expected for all indicators but any movement towards compliance is considered positive.

PRIMIS is able to provide a full and tailored service which includes access to a PINCER Specification and GP system searches for the PINCER indicators; IT support for the implementation of those searches within existing software solutions; a comparative analysis service that permits practices to benchmark themselves against local averages, or against themselves at an earlier point in time; and training and educational materials to enable primary care pharmacists to implement the PINCER intervention in GP practices in England. More information is available on the PINCER website.

Definitions of the PINCER hazardous prescribing indicators are provided in the table below.

Description	Denominator	Numerator (group at risk to the potentially hazardous prescribing event)
**Indicators associated with gastrointestinal bleeding**		
**Age ≥ 65 & NSAID**: prescription of an oral non-steroidal anti-inflammatory drug (NSAID), without co-prescription of an ulcer-healing drug, to a patient aged ≥65 years	Patients aged ≥65 years without co-prescription of an ulcer-healing drug (proton pump inhibitor (PPI) or H2 antagonist) in the 3 months leading up to the audit date	Patients in the group denominator AND prescribed an oral NSAID in the 3 months leading up to the audit date
**PU & NSAID**: prescription of an oral NSAID, without co-prescription of an ulcer healing drug, to a patient with a history of peptic ulceration	Patients aged ≥18 years with a Read code for peptic ulcer<br>or<br>GI bleed at least 3 months before audit date and not<br>prescribed an ulcer healing drug (PPI or H2 antagonist) within the 3 months leading up to the audit date	Patients in the group denominator AND prescribed an oral NSAID within the 3 months leading up to the audit date
**PU & antiplatelet**: prescription of an antiplatelet drug without co-prescription of an ulcer-healing drug, to a patient with a history of peptic ulceration	Patients aged ≥18 years with a Read code for peptic ulcer or<br>GI bleed at least 3 months before audit date and not<br>prescribed an ulcer healing drug (PPI or H2 antagonist) within the 3 months leading up to the audit date	Patients in the group denominator AND prescribed an antiplatelet drug (aspirin or clopidogrel or prasugrel or ticagrelor) within the 3 months leading up to the audit date
**Warfarin/DOAC & NSAID**: prescription of warfarin or DOAC in combination with an oral NSAID	Patients aged ≥18 years prescribed warfarin or a DOAC (apixaban or dabigatran or rivaroxaban or edoxaban) within the 3 months leading up to the audit date	Patients in the group denominator AND prescribed an oral NSAID within the 3 months leading up to the audit date
**Warfarin/DOAC & antiplatelet**: prescription of warfarin or DOAC and an antiplatelet drug in combination without coprescription of an ulcer-healing drug	Patients aged ≥18 years prescribed warfarin or DOAC without co-prescription of ulcer-healing drug (PPI or H2 antagonist) within the 3 months leading up to the audit date	Patients in the group denominator AND prescribed an antiplatelet drug (aspirin or clopidogrel or prasugrel or ticagrelor) within the 3 months leading up to the audit date and within 28 days of the warfarin/ DOAC prescription
**Aspirin & other antiplatelet**: prescription of aspirin in combination with another antiplatelet drug (without coprescription of an ulcer-healing drug)	Patients aged ≥18 years prescribed aspirin without coprescription of ulcer-healing drug (PPI or H2 antagonist) within the 3 months leading up to the audit date	Patients in the group denominator AND prescribed another antiplatelet drug (clopidogrel or prasugrel or ticagrelor) within the 3 months leading up to the audit date and within 28 days of the aspirin prescription
**Indicators associated with cautioned medication in other conditions (including heart failure, asthma and acute kidney injury)**		
**HF & NSAID**: prescription of an	Patients aged ≥18 years who have a	Patients in the group denominator AND prescribed an oral NSAID

oral NSAID to a patient with heart failure	diagnosis of heart failure at least 3 months before the audit date	within the 3 months leading up to the audit date
**Asthma & beta-blocker**: prescription of a non-selective beta-blocker to a patient with asthma	Patients aged ≥18 years with a Read code for asthma at least 3 months before audit date and no subsequent asthma resolved code during that time period	Patients in the group denominator AND prescribed a non-selective β-blocker within the 3 months leading up to the audit date
**CRF & NSAID**: prescription of an oral NSAID to a patient with eGFR <45	Patients aged ≥18 years with chronic renal failure: eGFR <45 at least 3 months before the audit date	Patients in the group denominator AND prescribed an oral NSAID within the 3 months leading up to the audit date
**Indicators associated blood test monitoring**		
**ACEI or loop diuretic, no blood tests**: patients aged 75 years and older who have been prescribed an angiotensin converting enzyme (ACE) inhibitor or a loop diuretic long term who have not had a computer-recorded check of their renal function and electrolytes in the previous 15 months	Patients aged ≥75 years prescribed an ACE inhibitor or a loop diuretic long-term i.e. first prescription for an ACE inhibitor or a loop diuretic at least 15 months prior to the audit date and at least one prescription (for the same drug) in the 6 months leading up to the audit date	Patients in the group denominator AND who have not had a computer-recorded check of their renal function and electrolytes within the previous 15 months leading up to the audit date
Patients receiving methotrexate for at least 3 months who have not had a recorded:<br>● Full blood count (FBC) within the previous 3 months (**Methotrexate and no FBC**)<br>● Liver function test (LFT) within the previous 3 months (**Methotrexate and no LFT**)	Patients aged ≥18 years with one or more prescriptions for methotrexate 3 to 6 months prior to the audit date and in the 3 months leading up to the audit date	Patients in the group denominator AND who have not had a computer-recorded:<br>● FBC within the 3 months leading up to the audit date<br>● LFT within the 3 months leading up to the audit date
**Lithium and no level recording**: patients receiving lithium for at least 3 months who have not had a recorded check of their lithium concentrations in the previous 3 months	Patients aged ≥18 years with one or more prescriptions for lithium recorded on computer 3 to 6 months prior to the audit date and in the 3 months leading up to the audit date	Patients in the group denominator AND who have not had a computer-recorded lithium level within the 3 months leading up to the audit date
**Amiodarone and no TFT**: patients receiving amiodarone for at least 6 months who have not had a thyroid function test (TFT) within the previous 6 months	Patients aged ≥18 years with one or more prescriptions for amiodarone 6 to 12 months prior to the audit date and in the 6 months leading up to the audit date	Patients in the group denominator AND who have not had a computer-recorded TFT within the 6 months leading up to the audit date

1. Avery AJ, Rodgers S, Cantrill JA, Armstrong S, Elliott R, Howard R, et al. Protocol for the PINCER trial: a cluster randomised trial comparing the effectiveness of a pharmacist-led IT-based intervention with simple feedback in reducing rates of clinically important errors in medicines management in general practices. Trials. 2009;10: 28.

2. Avery AJ, Rodgers S, Cantrill JA, Armstrong S, Cresswell K, Eden M, et al. A pharmacist-led information technology intervention for medication errors (PINCER): a multicentre, cluster randomised, controlled trial and cost-effectiveness analysis. Lancet. 2012;379: 1310–1319.

3. PRIMIS Team. PINCER National Rollout: Progress Report to NHS England and the AHSN Network. University of Nottingham; 2020 Jul. Available: https://www.nottingham.ac.uk/primis/documents/pincer/pincer-progress-report-july-2020.pdf

Supplementary Figures

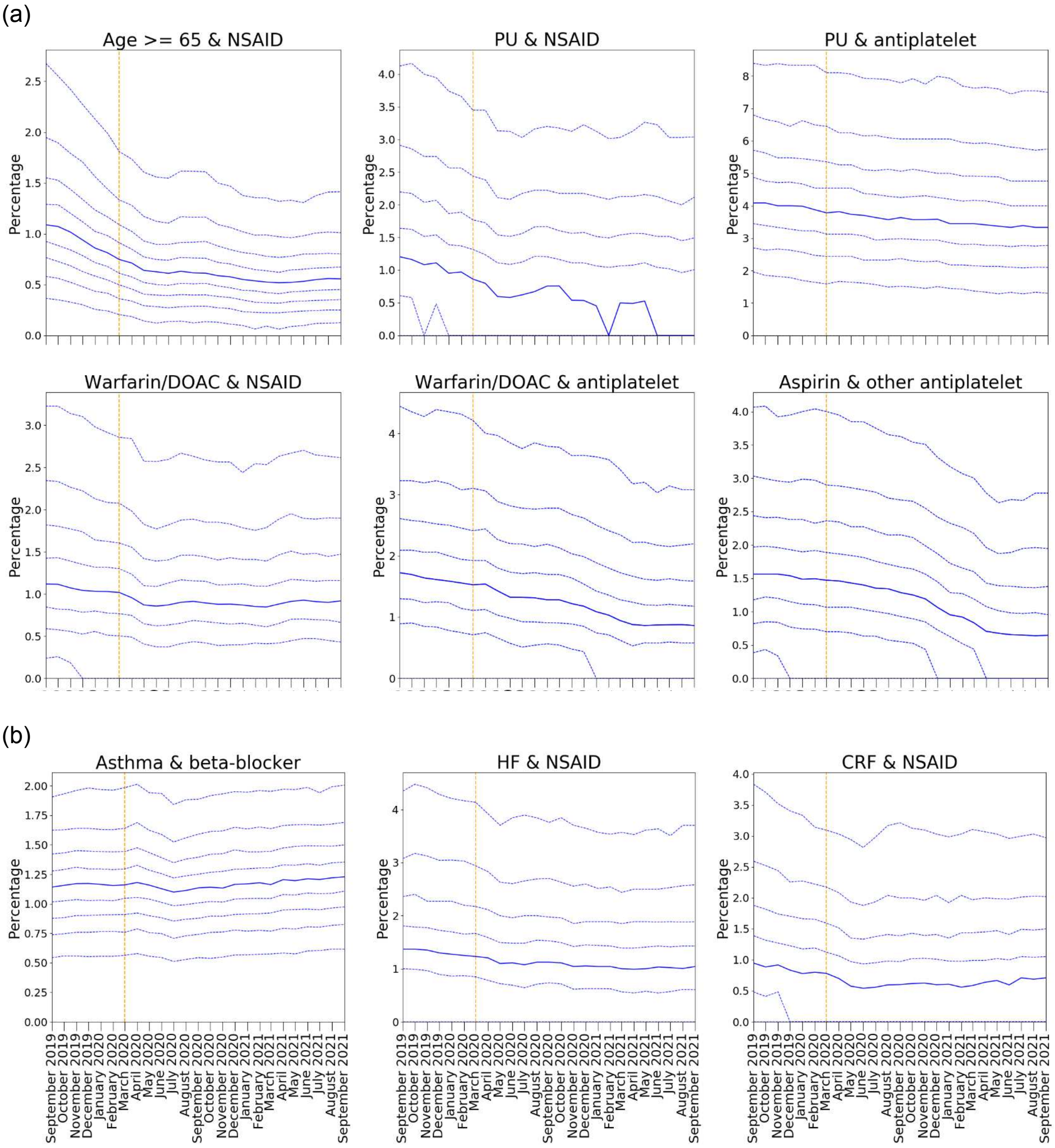

Supplementary Figure 1 - OpenSAFELY-TPP practice level decile plots for PINCER prescribing indicators, specifically in relation to (a) GI bleeding and (b) cautioned medications. All deciles are calculated across 2546 OpenSAFELY-TPP practices. The percentage of patients identified as at risk of potentially hazardous prescribing as measured by each indicator is reported for the period September 2019 to September 2021 (inclusive). The median percentage is displayed as a thick blue line and deciles are indicated by dashed blue lines. The month of national lockdown in England as a response to the onset of COVID-19 (March 2020) is highlighted with an orange dashed vertical line.

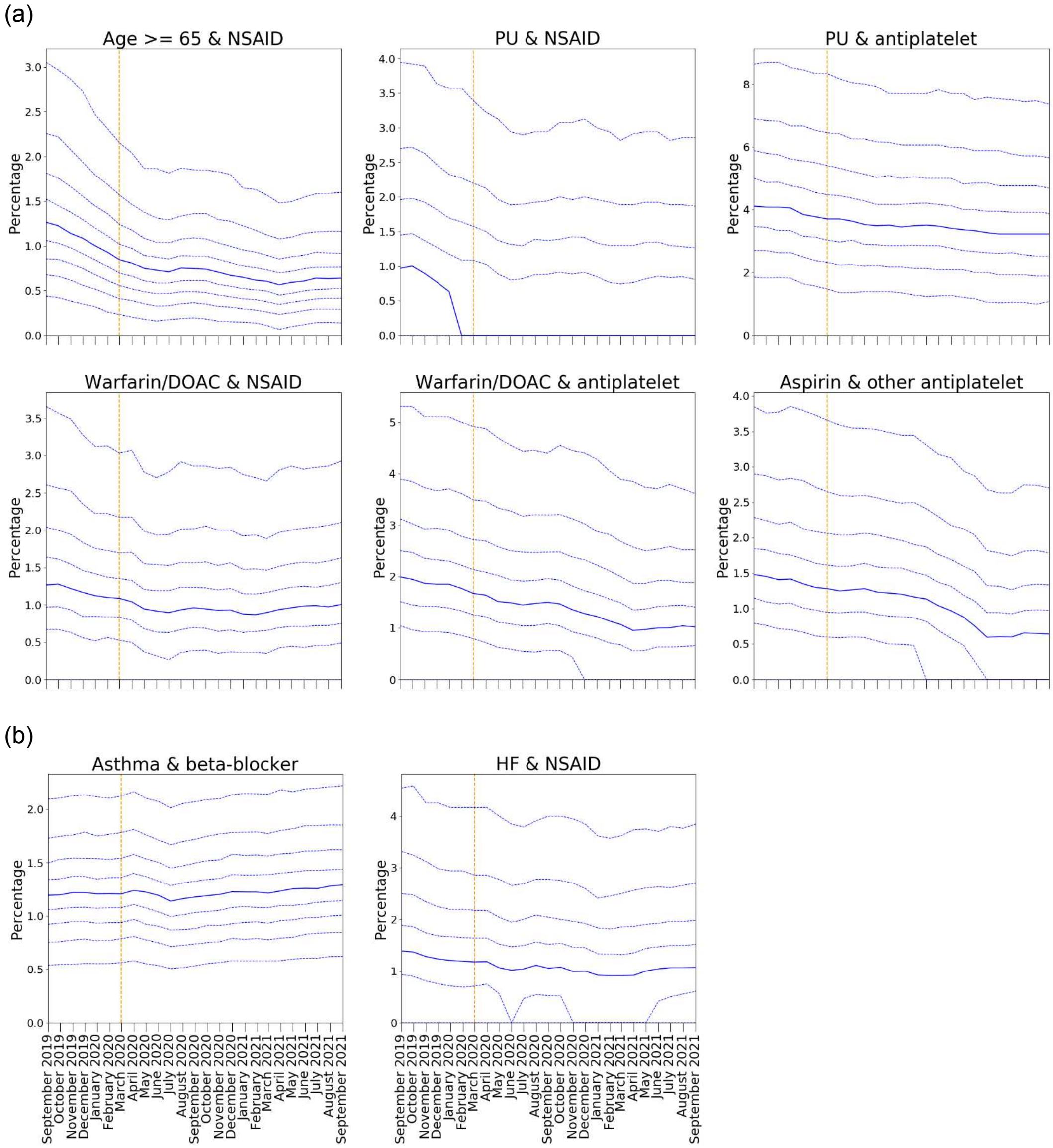

Supplementary Figure 2 - OpenSAFELY-EMIS practice level decile plots for PINCER prescribing indicators, specifically in relation to (a) GI bleeding and (b) cautioned medications. All deciles are calculated across 3821 OpenSAFELY-EMIS practices. The percentage of patients identified as at risk of potentially hazardous prescribing as measured by each indicator is reported for the period September 2019 to September 2021 (inclusive). The median percentage is displayed as a thick blue line and deciles are indicated by dashed blue lines. The month of national lockdown in England as a response to the onset of COVID-19 (March 2020) is highlighted with an orange dashed vertical line. Note that the CRF & NSAID indicator could not be implemented in OpenSAFELY-EMIS and therefore not shown.

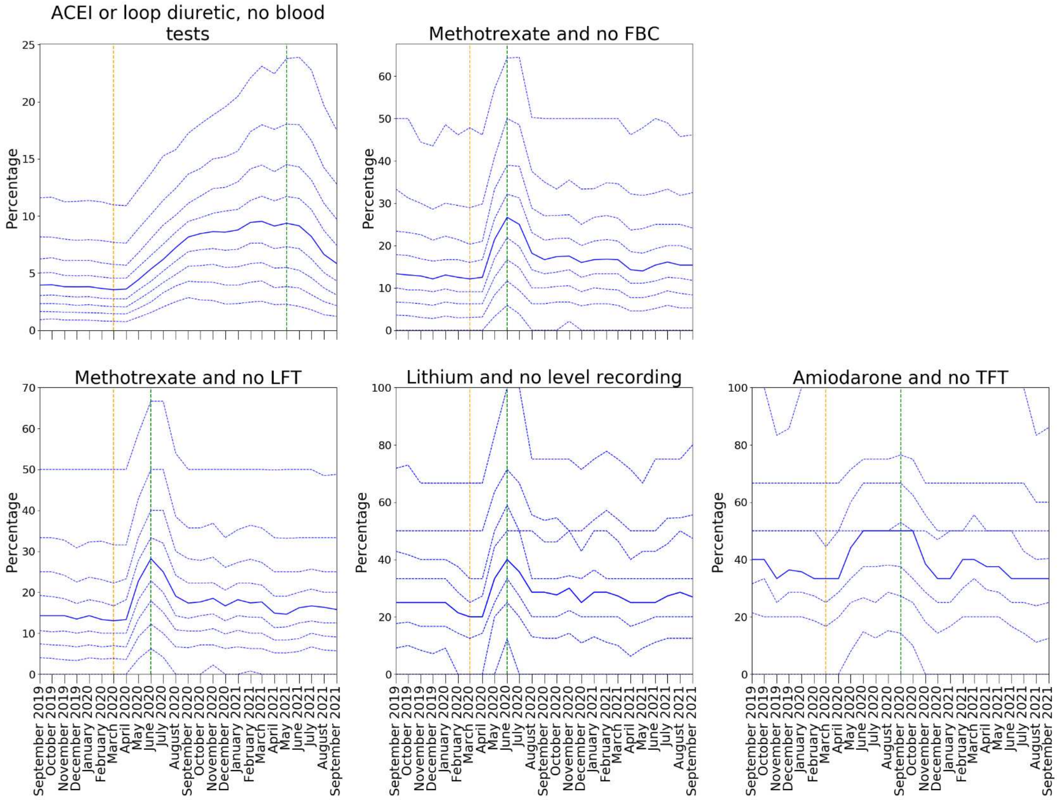

Supplementary Figure 3 - OpenSAFELY-TPP practice level decile plots for PINCER blood test monitoring indicators. All deciles are calculated across 2546 OpenSAFELY-TPP practices. The percentage of patients identified as at risk of potentially blood test monitoring as measured by each indicator is reported for the period September 2019 to September 2021 (inclusive). The median percentage is displayed as a thick blue line and deciles are indicated by dashed blue lines. The month of national lockdown in England as a response to the onset of COVID-19 (March 2020) is highlighted with an orange dashed vertical line. The monitoring window, as measured from the onset of COVID-19, for each indicator is shown by a green dashed vertical line.

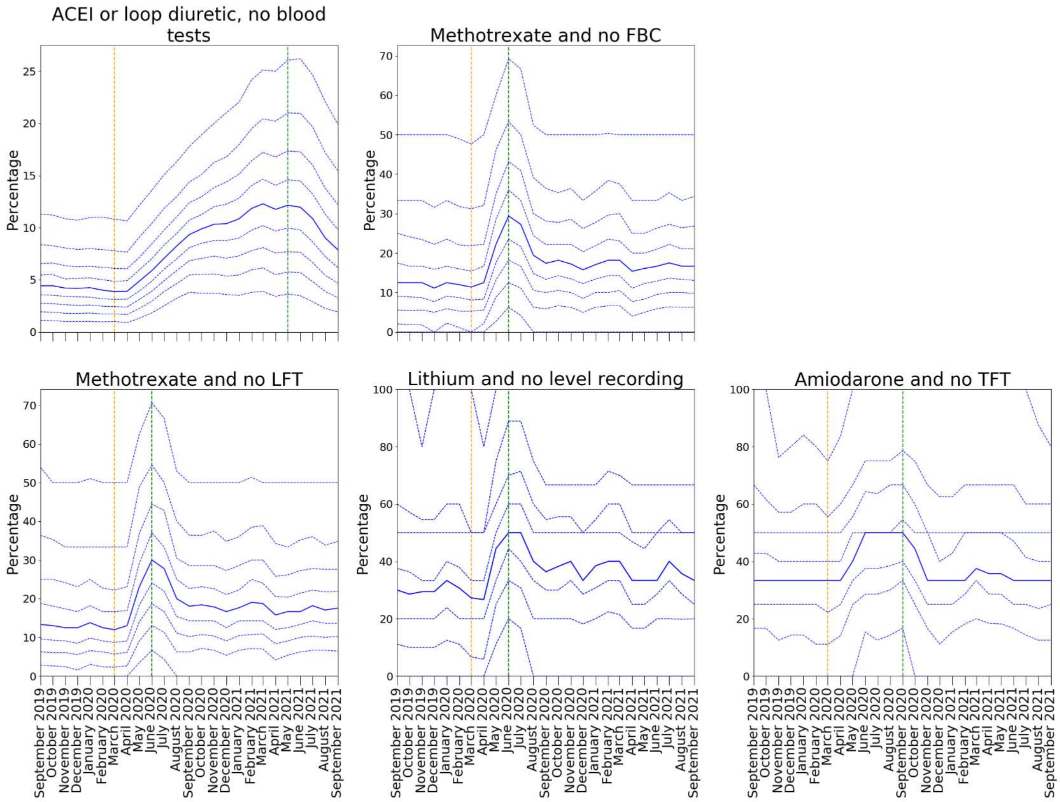

Supplementary Figure 4 - OpenSAFELY-EMIS practice level decile plots for PINCER blood test monitoring indicators. All deciles are calculated across 3821 OpenSAFELY-EMIS practices. The percentage of patients identified as at risk of potentially blood test monitoring as measured by each indicator is reported for the period September 2019 to September 2021 (inclusive). The median percentage is displayed as a thick blue line and deciles are indicated by dashed blue lines. The month of national lockdown in England as a response to the onset of COVID-19 (March 2020) is highlighted with an orange dashed vertical line. The monitoring window, as measured from the onset of COVID-19, for each indicator is shown by a green dashed vertical line.

Supplementary Table 1	Indicator rates for PINCER hazardous prescribing indicators (in 2546 OpenSAFELY-TPP practices only): Q1 2020/2021 percentages and cumulative results between September 2019 and September 2021. Mean values calculated at the practice-level.											
	OpenSafely											
	Point in time			Cumulative results (Sept 2019-Sept 2021)								
Indicator	**Q1 median proportion 2020**	**Q1 median proportion 2021**	**Q1 2021 - Q1 2020**	**Numerator**	**Denominator**	**%**	**Numerator (%)**	**Number of hazardous prescribing events**	**Ratio of events to patients**	**Number of practices with at least one hazardous prescribing event**	**% of total number of practices**	**Number of practices with at least one hazardous prescribing event (% of total number of practices)**
**Indicators associated with gastroi**	-	-	-	235912	4743819	4.97	235912 (4.97%)	-	-	2538	-	-
**Age ≥ 65 & NSAID:** prescription of an oral non-steroidal anti-inflammatory drug (NSAID), without co-prescription of an ulcer-healing drug, to a patient aged ≥65 years	1.02	0.67	-0.35	142278	4030402	3.53	142278 (3.53%)	633881	4.46	2524	99.14	2524 (99.14%)
**PU & NSAID:** prescription of an oral NSAID, without co-prescription of an ulcer healing drug, to a patient with a history of peptic ulceration	1.41	1.14	-0.27	14993	283554	5.29	14993 (5.29%)	57124	3.81	2369	93.05	2369 (93.05%)
**PU & antiplatelet:** prescription of an antiplatelet drug without co-prescription of an ulcer-healing drug, to a patient with a history of peptic ulceration	4.26	3.9	-0.36	17511	283554	6.18	17511 (6.18%)	193083	11.03	2382	93.56	2382 (93.56%)
**Warfarin/DOAC & NSAID:** prescription of warfarin or DOAC in combination with an oral NSAID	1.36	1.16	-0.20	36318	844996	4.3	36318 (4.3%)	163730	4.51	2499	98.15	2499 (98.15%)
**Warfarin/DOAC & antiplatelet:** prescription of warfarin or DOAC and an antiplatelet drug in combination without coprescription of an ulcer-healing drug	2	1.45	-0.55	21414	552859	3.87	21414 (3.87%)	128221	5.99	2430	95.44	2430 (95.44%)
**Aspirin & other antiplatelet:** prescription of aspirin in combination with another antiplatelet drug (without coprescription of an ulcer-healing drug)	1.83	1.29	-0.54	16206	628228	2.58	16206 (2.58%)	116318	7.18	2280	89.55	2280 (89.55%)

Supplementary Table 1	Indicator rates for PINCER hazardous prescribing indicators (in 2546 OpenSAFELY-TPP practices only): Q1 2020/2021 percentages and cumulative results between September 2019 and September 2021. Mean values calculated at the practice-level.											
	OpenSafely											
	Point in time			Cumulative results (Sept 2019-Sept 2021)								
Indicator	**Q1 median proportion 2020**	**Q1 median proportion 2021**	**Q1 2021 - Q1 2020**	**Numerator**	**Denominator**	**%**	**Numerator (%)**	**Number of hazardous prescribing events**	**Ratio of events to patients**	**Number of practices with at least one hazardous prescribing event**	**% of total number of practices**	**Number of practices with at least one hazardous prescribing event (% of total number of practices)**
**Indicators associated with gastroi**	-	-	-	235912	4743819	4.97	235912 (4.97%)	-	-	2538	-	-
**Indicators associated with cautioned medication in other conditions (including heart failure, asthma and acute kidney injury)**	-	-	-	101633	3338331	3.04	101633 (3.04%)	-	-	2532	-	-
**Asthma & beta-blocker:** prescription of a non-selective beta-blocker to a patient with asthma	1.23	1.25	0.02	75833	2744049	2.76	75833 (2.76%)	772431	10.19	2525	99.18	2525 (99.18%)
**HF & NSAID:** prescription of an oral NSAID to a patient with heart failure	1.75	1.44	-0.31	13378	317170	4.22	13378 (4.22%)	83559	6.25	2272	89.24	2272 (89.24%)
**CRF & NSAID:** prescription of an oral NSAID to a patient with eGFR <45	1.27	1.12	-0.15	14558	476925	3.05	14558 (3.05%)	68384	4.7	2217	87.08	2217 (87.08%)
**Indicators associated blood test monitoring**	-		-	466522	1477986	31.56	466522 (31.56%)	-	-	2531	-	-
**ACEI or loop diuretic, no blood tests:** patients aged 75 years and older who have been prescribed an angiotensin converting enzyme (ACE) inhibitor or a loop diuretic long term who have not had a computer-recorded check of their renal function and electrolytes in the previous 15 months	5.08	11	5.92	356896	1363003	26.18	356896 (26.18%)	2070891	5.8	2521	99.02	2521 (99.02%)
**Methotrexate and no FBC:** patients receiving methotrexate for at least 3 months who have not had a recorded full blood count (FBC) within the previous 3 months	18.52	22.11	3.59	72265	104241	69.32	72265 (69.32%)	371960	5.15	2511	98.63	2511 (98.63%)

Supplementary Table 1	Indicator rates for PINCER hazardous prescribing indicators (in 2546 OpenSAFELY-TPP practices only): Q1 2020/2021 percentages and cumulative results between September 2019 and September 2021. Mean values calculated at the practice-level.											
	OpenSafely											
	Point in time			Cumulative results (Sept 2019-Sept 2021)								
Indicator	**Q1 median proportion 2020**	**Q1 median proportion 2021**	**Q1 2021 - Q1 2020**	**Numerator**	**Denominator**	**%**	**Numerator (%)**	**Number of hazardous prescribing events**	**Ratio of events to patients**	**Number of practices with at least one hazardous prescribing event**	**% of total number of practices**	**Number of practices with at least one hazardous prescribing event (% of total number of practices)**
**Indicators associated with gastroi**	-	-	-	235912	4743819	4.97	235912 (4.97%)	-	-	2538	-	-
**Methotrexate and no LFT:** patients receiving methotrexate for at least 3 months who have not had a recorded liver function test (LFT) within the previous 3 months	19.73	22.78	3.05	73305	104241	70.32	73305 (70.32%)	385483	5.26	2511	98.63	2511 (98.63%)
**Lithium and no level recording:** patients receiving lithium for at least 3 months who have not had a recorded check of their lithium concentrations in the previous 3 months	27.47	32.88	5.41	18118	20626	87.84	18118 (87.84%)	114354	6.31	2422	95.13	2422 (95.13%)
**Amiodarone and no TFT:** patients receiving amiodarone for at least 6 months who have not had a thyroid function test (TFT) within the previous 6 months	38.47	40.32	1.85	19019	24524	77.55	19019 (77.55%)	122358	6.43	2406	94.5	2406 (94.5%)

Supplementary Table 2	Indicator rates for PINCER hazardous prescribing indicators (in 3821 OpenSAFELY-EMIS practices only): Q1 2020/2021 percentages and cumulative results between September 2019 and September 2021. Mean values calculated at the practice-level. No results provided for CRF & NSAID as this indicator could not be implemented in OpenSAFELY-EMIS.											
	OpenSafely											
	Point in time			Cumulative results (Sept 2019-Sept 2021)								
Indicator	**Q1 median proportion 2020**	**Q1 median proportion 2021**	**Q1 2021 - Q1 2020**	**Numerator**	**Denominator**	**%**	**Numerator (%)**	**Number of hazardous prescribing events**	**Ratio of events to patients**	**Number of practices with at least one hazardous prescribing event**	**% of total number of practices**	**Number of practices with at least one hazardous prescribing event (% of total number of practices)**
**Indicators associated with gastroi**	-	-	-	315932	6137856	5.15	315932 (5.15%)	-	-	3797	-	-
**Age ≥ 65 & NSAID:** prescription of an oral non-steroidal anti-inflammatory drug (NSAID), without co-prescription of an ulcer-healing drug, to a patient aged ≥65 years	1.16	0.79	-0.37	192209	5176605	3.71	192209 (3.71%)	885005	4.6	3780	98.93	3780 (98.93%)
**PU & NSAID:** prescription of an oral NSAID, without co-prescription of an ulcer healing drug, to a patient with a history of peptic ulceration	1.25	1.01	-0.24	17096	394664	4.33	17096 (4.33%)	65853	3.85	3432	89.82	3432 (89.82%)
**PU & antiplatelet:** prescription of an antiplatelet drug without co-prescription of an ulcer-healing drug, to a patient with a history of peptic ulceration	4.23	3.81	-0.42	23903	394664	6.06	23903 (6.06%)	258187	10.8	3561	93.2	3561 (93.2%)
**Warfarin/DOAC & NSAID:** prescription of warfarin or DOAC in combination with an oral NSAID	1.41	1.19	-0.22	47783	1070121	4.47	47783 (4.47%)	220850	4.62	3694	96.68	3694 (96.68%)
**Warfarin/DOAC & antiplatelet:** prescription of warfarin or DOAC and an antiplatelet drug in combination without coprescription of an ulcer-healing drug	2.26	1.67	-0.59	31161	697006	4.47	31161 (4.47%)	179827	5.77	3638	95.21	3638 (95.21%)
**Aspirin & other antiplatelet:** prescription of aspirin in combination with another antiplatelet drug (without coprescription of an ulcer-healing drug)	1.67	1.2	-0.47	20721	842087	2.46	20721 (2.46%)	147539	7.12	3497	91.52	3497 (91.52%)
**Indicators associated with cautioned medication in other conditions (including heart failure, asthma and acute kidney injury)**	-	-	-	126954	4256356	2.98	126954 (2.98%)	-	-	3789	-	-

Supplementary Table 2	Indicator rates for PINCER hazardous prescribing indicators (in 3821 OpenSAFELY-EMIS practices only): Q1 2020/2021 percentages and cumulative results between September 2019 and September 2021. Mean values calculated at the practice-level. No results provided for CRF & NSAID as this indicator could not be implemented in OpenSAFELY-EMIS.											
	OpenSafely											
	Point in time			Cumulative results (Sept 2019-Sept 2021)								
Indicator	**Q1 median proportion 2020**	**Q1 median proportion 2021**	**Q1 2021 - Q1 2020**	**Numerator**	**Denominator**	**%**	**Numerator (%)**	**Number of hazardous prescribing events**	**Ratio of events to patients**	**Number of practices with at least one hazardous prescribing event**	**% of total number of practices**	**Number of practices with at least one hazardous prescribing event (% of total number of practices)**
**Indicators associated with gastroi**	-	-	-	315932	6137856	5.15	315932 (5.15%)	-	-	3797	-	-
**Asthma & beta-blocker:** prescription of a non-selective beta-blocker to a patient with asthma	1.3	1.32	0.02	109456	3902537	2.8	109456 (2.8%)	1125619	10.28	3782	98.98	3782 (98.98%)
**HF & NSAID:** prescription of an oral NSAID to a patient with heart failure	1.68	1.43	-0.25	17656	418611	4.22	17656 (4.22%)	105402	5.97	3350	87.67	3350 (87.67%)
**CRF & NSAID:** prescription of an oral NSAID to a patient with eGFR <45	-	-	-	-	-	-	- (-%)	-	-	-	-	-
**Indicators associated blood test monitoring**	-	-	-	635687	1880968	33.8	635687 (33.8%)	-	-	3798	-	-
**ACEI or loop diuretic, no blood tests:** patients aged 75 years and older who have been prescribed an angiotensin converting enzyme (ACE) inhibitor or a loop diuretic long term who have not had a computer-recorded check of their renal function and electrolytes in the previous 15 months	5.2	12.9	7.7	493691	1732592	28.49	493691 (28.49%)	2935654	5.95	3782	98.98	3782 (98.98%)
**Methotrexate and no FBC:** patients receiving methotrexate for at least 3 months who have not had a recorded full blood count (FBC) within the previous 3 months	18.73	23.15	4.42	92237	133801	68.94	92237 (68.94%)	459580	4.98	3767	98.59	3767 (98.59%)
**Methotrexate and no LFT:** patients receiving methotrexate for at least 3 months who have not had a recorded liver function test (LFT) within the previous 3 months	19.54	23.6	4.06	93179	133801	69.64	93179 (69.64%)	470010	5.04	3767	98.59	3767 (98.59%)

Supplementary Table 2	Indicator rates for PINCER hazardous prescribing indicators (in 3821 OpenSAFELY-EMIS practices only): Q1 2020/2021 percentages and cumulative results between September 2019 and September 2021. Mean values calculated at the practice-level. No results provided for CRF & NSAID as this indicator could not be implemented in OpenSAFELY-EMIS.										
	OpenSafely										
	Point in time			Cumulative results (Sept 2019-Sept 2021)							
Indicator	**Q1 median proportion 2020**	**Q1 median proportion 2021**	**Q1 2021 - Q1 2020**	**Numerator**	**Denominator**	**%**	**Numerator (%)**	**Number of hazardous prescribing events**	**Ratio of events to patients**	**Number of practices with at least one hazardous prescribing event**	**% of total number of practices**	**Number of practices with at least one hazardous prescribing event (% of total number of practices)**
**Indicators associated with gastroi**	-	-	-	315932	6137856	5.15	315932 (5.15%)	-	-	3797	-	-
**Lithium and no level recording:** patients receiving lithium for at least 3 months who have not had a recorded check of their lithium concentrations in the previous 3 months	34.28	42.32	8.04	22546	24830	90.8	22546 (90.8%)	167281	7.42	3490	91.34	3490 (91.34%)
**Amiodarone and no TFT:** patients receiving amiodarone for at least 6 months who have not had a thyroid function test (TFT) within the previous 6 months	34.63	38.49	3.86	27249	35401	76.97	27249 (76.97%)	165510	6.07	3587	93.88	3587 (93.88%)
